# Do Different Species of* Sargassum* in Haizao Yuhu Decoction Cause Different Effects in a Rat Goiter Model?

**DOI:** 10.1155/2019/5645620

**Published:** 2019-01-06

**Authors:** Dianna Liu, Feng Chen, Xue Yu, Linlin Xiu, Haiyan Liu, Shaohong Chen, Jie Gao, Chen Zhang, Na Li, Cheng He, Gansheng Zhong

**Affiliations:** Beijing University of Chinese Medicine, No. 11 East Road, North 3rd Ring Road, Chaoyang District, Beijing 100029, China

## Abstract

*Sargassum *species combined with* Glycyrrhiza uralensis* is a famous herbal pair in traditional Chinese medicine, as one of the so-called “eighteen antagonistic medicaments.” In the Chinese Pharmacopoeia, two different species of* Sargassum*,* Sargassum pallidum* and* Sargassum fusiforme*, are recorded but they are not clearly differentiated in clinical use. In this study, we aimed to determine whether the two species of* Sargassum* could result in different effects when combined with* G. uralensis* in Haizao Yuhu Decoction (HYD), which is used for treating thyroid-related diseases, especially goiter. HYD containing* S. pallidum* or* S. fusiforme* was administered to rats with propylthiouracil-induced goiter. After 4 weeks, pathological changes in the thyroid tissue and the relative thyroid weight indicated that HYD containing* S. pallidum *or* S. fusiforme *protected thyroid tissues from propylthiouracil damage. Neither species increased the propylthiouracil-induced decrease in serum levels of thyroid hormones. However, there were some differences in their actions, and only HYD containing* S. fusiforme* abated the propylthiouracil-induced elevation of serum thyroid-stimulating hormone levels and activated thyroglobulin mRNA expression.

## 1. Introduction

Traditional Chinese medicine (TCM) is one of the most popular complementary and alternative medicine modalities worldwide. In “Confucians' Duties to Their Parents,” one of the most famous clinical monographs of TCM, there is a widely circulated song tactics called “eighteen antagonistic medicaments.” The herb pair* Sargassum *and* Glycyrrhiza uralensis* (GanCao, GC) belongs to the “eighteen antagonistic medicaments,” which means that they are incompatible and theoretically should not be applied simultaneously. However, this herb pair has been used for at least 1,000 years and has been described in “Taiping Holy Prescriptions for Universal Relief,” “Summary of Surgical Medicine,” and the “Pharmacopoeia of the People's Republic of China (Chinese Pharmacopoeia)” [1]. Haizao Yuhu Decoction (HYD) contains this herb pair and is widely known for its efficacy in treating thyroid-related diseases, especially goiter, and has been described in “Summary of Surgical Medicine” of the Chinese Ming Dynasty. Currently, HYD is also widely used to treat mammary gland hyperplasia [2], ovarian cysts [3], and hypertrophic pharyngitis [4] based on the TCM mechanism of dissipating “phlegm” and removing mass.

In the Chinese Pharmacopoeia, two different species of* Sargassum*,* Sargassum pallidum* and* Sargassum fusiforme,* have been described and numerous ancient Chinese medical monographs also report these two species as Hai HaoZi (HHZ) and Yang Qi Cai (YQC), respectively. They are compositionally different [5, 6]. However, whether the two different species of* Sargassum *cause different effects when coapplied with GC simultaneously in HYD has not been reported. No differences have been previously reported between the antigoiter effects of HHZ and YQC application in HYD, even at twice the high-dose limit recommended in the Chinese Pharmacopoeia [7]. However, the herb pair is clinically effective over a large dose range [8, 9]. In this study, we investigated the differences between HHZ and YQC coadministered with GC to rats with propylthiouracil- (PTU-) induced goiter at four times the high-dose limit in HYD as specified by the Chinese Pharmacopoeia.

## 2. Materials and Methods

### 2.1. Preparation of HYD

HYD contains 12 Chinese herbs, listed in Table 1. We prepared two types of HYD, one containing HHZ and the other containing YQC. All herbs used in the study were purchased from Beijing Shuangqiao Co., Ltd. (Beijing, China) and Anhui Tongling Co., Ltd. (Tongling, China) and authenticated by Professor Li (School of Basic Medical Science, Beijing University of Chinese Medicine).

The mixtures were boiled for a further 60 min after soaking for at least 2 h in distilled water at a ratio of 1/10 (g/ml). Then, the residue was boiled again for a further 30 min and supplemented with a ratio of 1/8 (g/ml) distilled water. Finally, the two portions of aqueous extracts were pooled together and concentrated to a density of 1.602 g/ml (calculated by the ratio of body surface area between humans and rats) by means of heating evaporation.

We determined the contents of several main active components in the two different kinds of HYD using high-performance liquid chromatography (HPLC), according to the Chinese Pharmacopoeia and methods described in our previous study [10]. Data analysis was performed in triplicate (Table 2). Compared with the HYD-H group, the HYD-Y group showed significant increase in the levels of hesperidin, osthole, and forsythin, and significant decrease in the levels of peimine and peiminine (*P*<0.05, Table 2). There were no differences between the HYD-H and HYD-Y group in the levels of liquiritin, glycyrrhizic acid, and ferulic acid.

### 2.2. Chemical Reagents

PTU was obtained from Zhaohui Pharmaceutical Technology Corporation (Shanghai, China). Euthyrox was purchased from MerkKGaA (Darmstadt, Germany).

### 2.3. Animals

A total of 100 Wistar rats (number of male rats=50; number of female rats=50; weight 180-220 g) were purchased from Charles River Laboratories, Beijing, China (Certificate of Conformity: SCXK (Beijing) 2012-0001). All the animals were housed under controlled quarters with a constant temperature (22±2°C), humidity (50±10%) and light illumination (12 h/d) (Certificate of Conformity: SCXK (Beijing) 2011-0024) with free access to food and water. All animal experiments and procedures were approved by the Animal Ethics Committee of the Beijing University of Chinese Medicine on September 27, 2017, and the project identification code was BUCM-4-2016080304-3004. All the animals were maintained in accordance with the guidelines outlined by the Chinese legislation on the ethical use and care of laboratory animals. Moreover, all efforts were made to minimize animal suffering and the number of animals used to produce reliable data.

### 2.4. Modeling and Grouping

After 5 days of acclimatization, one hundred rats were randomly divided into five groups (n = 20/group): control (Con), model (Mod), Euthyrox (Eut), HYD containing HHZ (HYD-H), and HYD containing YQC (HYD-Y). All the animals were randomized based on body weight, according to random numbers generated by Excel 2010 (Microsoft Corporation, Washington, USA).

All the animals except those in the Con group received PTU (0.01 g/kg/d, intragastric) for 14 days to induce the goitrous model according to previous reports [7, 11]. On day 15, blood sample was collected from all animals in the Con group and 20 rats in the other groups, which were chosen randomly to prove that the model was established. Blood samples (2 mL) were then collected from the jugular vein following light ether anesthesia, and the serum was obtained to analyze triiodothyronine (T3), thyroxine (T4), and thyroid-stimulating hormone (TSH) levels. Then, all the rats were given the drug treatment for 28 days. The Con and Mod groups received normal saline (10 ml/kg/d, intragastric). The Eut group was given Euthyrox (0.02 mg/kg/d, intragastric). Group HYD-H and Group HYD-Y received HYD containing HHZ or YQC (16.02 g/kg/d, intragastric). During the treatment period, to maintain the goitrous model, all the animals except those in the Con group were administered PTU (0.01 g/kg/d, intragastric) every two days, and the Con group was treated with distilled water (10 ml/kg/d, intragastric). All the rats received liquid at a dose of 1 ml/100 g each time.

### 2.5. General Observations

Clinical signs and mortality were recorded twice per day (before and after treatment) throughout the study period. The body weight and rectal temperature of each rat were measured at the initiation of the study and once a week during the backing period. At the end of the study, thyroid weights were noted, and the relative thyroid weight was calculated relative to body weight.

### 2.6. Biochemical Assays

At the end of the treatments, before being sacrificed, the animals were deeply anesthetized with pentobarbital sodium (50mg/kg). Blood samples were collected from the abdominal artery and immediately centrifuged at a speed of 3000 r/min for 15 min at 4°C to obtain serum, which was stored at -80°C for biochemical analysis. The serum levels of T3, T4, free T3 (FT3), free T4 (FT4), TSH, thyrotropin-releasing hormone (THR), thyrotropin-releasing hormone receptor (TSHR), TSHR antibody (TRAb), and thyroglobulin (Tg) were determined by radioimmunoassay using an automatic radioimmunocounter (XH-6020, CNNC XI'AN NUCLEAR INSTRUMENT FACTORY, China). The serum levels of malondialdehyde (MDA), superoxide dismutase (SOD), and glutathione peroxidase (GSH-PX) were determined by colorimetry using a fully automated biochemical analyzer (BS-420, Mindray, China). All the commercially available test kits were purchased from Beijing Sino-UK Institute of Biological Technology (Beijing, China).

### 2.7. Histopathology

Both sides of the thyroid tissues were completely stripped after all the rats had been sacrificed. One side of the thyroid tissue was fixed with 4%(w/v) paraformaldehyde, followed by dehydration in graded ethanol and embedding in paraffin. Then, several 4-*μ*m-thick tissue sections were prepared and stained with hematoxylin and eosin (H&E) and observed using an optical microscope. The other thyroid side was immediately stored in liquid nitrogen at -80°C for real-time PCR analysis.

### 2.8. Real-Time Quantitative Polymerase Chain Reaction (PCR)

Six thyroid tissues were randomly selected from each group for real-time quantitative PCR. Total RNA was isolated from thyroid tissues using TRIzol agent (Invitrogen, USA). RNA yields and purity were assessed via spectrophotometric analysis (Spectronic Unicam, USA). Reverse transcriptase reactions were performed in a reaction mixture of 4 *μ*l 5x Reaction Buffer, 2 *μ*l dNTP Mix(10 mM), 1 *μ*l Oligo (dT) (100 *μ*M), 1 *μ*l RiboLock RNase inhibitor (20 U/*μ*lL), 1 *μ*l RevertAid RT (200 U/*μ*lL), 3 *μ*g total RNA, and nuclease-free water to a final volume of 20 *μ*l. The agent used for reverse transcriptase reactions came from a Thermo Scientific RevertAid First Strand cDNA Synthesis Kit. Then, 130 *μ*l double-distilled water was added in to the rReverse transcriptase production. The reaction mixtures were incubated first at 42°C for 60 min and then at 70°C for 5 min. Real-time PCR for each cDNA was performed in triplicate in a 20-*μ*l reaction mixture containing 5 *μ*l cDNA, 0.5 *μ*l each forward and reverse primer (both 10 mM), 4 *μ*l double-distilled water, and 10 *μ*l UltraSYBR Mixture (Applied CWBIO, China). Reactions were incubated in a C1000™ Thermal Cycler with a CFX96™ Real-Time SystermSystem (Bio-Rad, USA) at 95°C for 10 min, followed by 40 cycles of 95°C for 10 s and 55°C or 60°C (55°C for *β*-actin, TPO, and Tg; 60°C for NIS, Fas, and Bcl-2) for 30 s. The relative quantitative levels of individual cDNAs were calculated using the 2-(∆∆Ct) method. All the primer sequences are listed in Table 3.

### 2.9. Statistical Analysis

The results are presented as the mean ± standard deviation from 3 independent experiments at least. All statistical analyses were performed using SPSS software version 20.0 for Windows (SPSS Inc., Chicago, IL, USA). P<0.05 was considered statistically significant. One-way analysis of variance was used to examine the differences between groups.

## 3. Results

### 3.1. Rat Goiter Model

**Figure 1:**
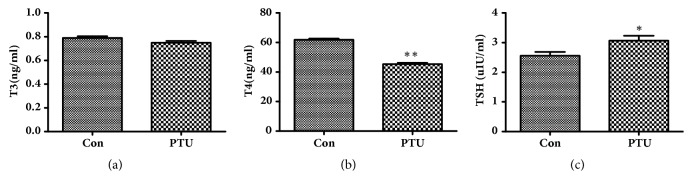
**Serum levels of triiodothyronine (T3), thyroxine (T4), and thyroid-stimulating hormone (TSH) after propylthiouracil (PTU) administration for 2 weeks.** (a) After receiving PTU for 2 weeks, serum levels of T3 decreased, although there was no statistical significance. (b) PTU administration for 2 weeks significantly reduced the serum levels of T4. (c) PTU administration for 2 weeks significantly increased the serum levels of TSH. **∗**P<0.05 and **∗∗**P<0.01, by one-way analysis of variance.

PTU administration for 2 weeks significantly reduced the serum levels of T4 (*P*<0.01, Figure 1) and increased those of TSH (*P*<0.05, Figure 1) in the rat model.

### 3.2. Rectal Temperature

**Figure 2:**
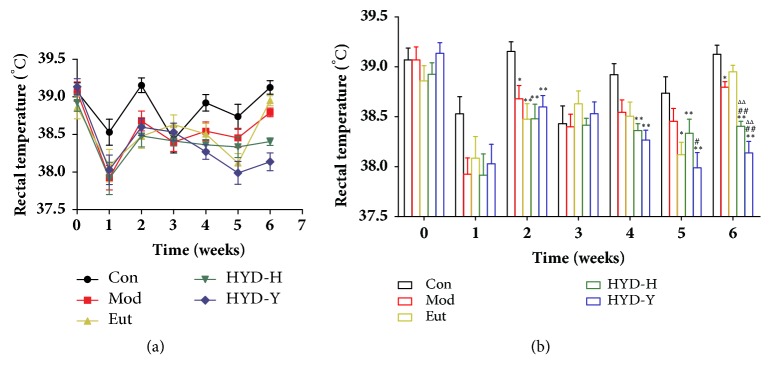
**Rectal temperature of rats in all groups.** (a) and (b) After receiving PTU for 2 weeks, the rectal temperature of all goitrous model rats was significantly decreased. Then, after drug treatment for 4 weeks, the rectal temperature of PTU-induced goiter rats could be significantly decreased by the administration of HYD-H and HYD-Y. **∗**P<0.05 and **∗∗**P<0.01 versus Con; #P<0.05 and ##P<0.01 versus Mod; ΔP<0.05 and ΔΔPP<0.01 versus Eut; $P<0.05 and $$P<0.01 versus HYD-H, by one-way analysis of variance.

PTU administration for 2 weeks significantly reduced the rectal temperature of all model rats (*P*<0.05, Figure 2), and there were no differences among the Mod, Eut, HYD-H, and HYD-Y groups. Similarly, HYD-H and HYD-Y treatments for 4 weeks significantly reduced the rectal temperature compared to the Con, model, and Eutyroxin-treated rats (*P*<0.01, Figure 2). However, no significant difference between the rectal temperature of the HYD-H and HYD-Y groups was observed.

### 3.3. Body Weight

**Figure 3:**
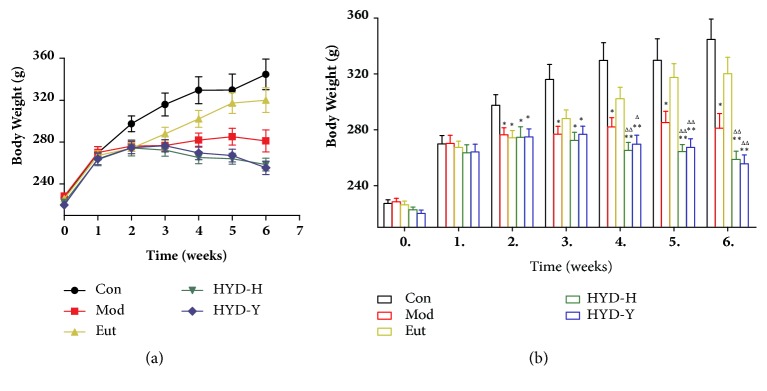
**Body weight of rats in all groups.** (a) and (b) After receiving PTU for 2 weeks, the body weight of all goitrous model rats was significantly decreased. Then, after drug treatment for 4 weeks, the body weight of PTU-induced goiter rats could be decreased by the administration of HYD-H and HYD-Y. **∗**P<0.05 and **∗∗**P<0.01 versus Con; #P<0.05 and ##P<0.01 versus Mod; ΔP<0.05 and ΔΔPP<0.01 versus Eut; $P<0.05 and $$P<0.01 versus HYD-H, by one-way analysis of variance.

PTU administration for 2 weeks significantly reduced the body weight of all model rats (*P*<0.05, Figure 3), and there were no differences among the Mod, Eut, HYD-H, and HYD-Y groups. During the drug treatment, the body weights of the control and Eutyroxin-treated rats gradually increased, those of the untreated model rats remained stable, and those of the HYD-H and HYD-Y rats decreased (Figure 3). After drug treatment for 28 days, body weights of the HYD-H- and HYD-Y-treated rats were significantly lower than those of the control and Eutyroxin-treated rats were (*P*<0.01, Figure 3). However, there was no significant difference among body weights of the Mod, HYD-H, and HYD-Y groups.

### 3.4. Absolute and Relative Thyroid Weights

**Figure 4:**
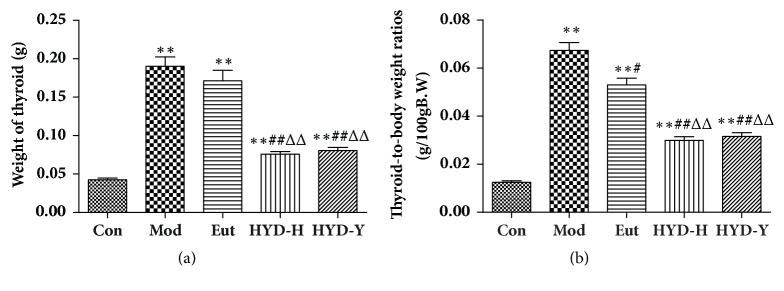
**Thyroid weight and relative thyroid weight of rats in all groups after drug treatment for 28 days.** (a) Elevated thyroid weight of goiter rats could be efficiently decreased by the administration of HYD-H and HYD-Y. (b) Elevated relative thyroid weight of goiter rats could be efficiently decreased by the administration of HYD-H and HYD-Y. **∗**P<0.05 and **∗∗**P<0.01 versus Con; #P<0.05 and ##P<0.01 versus Mod; ΔP<0.05, and ΔΔPP<0.01 versus Eut; $P<0.05 and $$P<0.01 versus HYD-H, by one-way analysis of variance.

After drug treatment for 28 days, compared with control rats, those in all the other treatment groups showed significantly increased absolute and relative thyroid weights (*P*<0.01, Figure 4). Compared with model and Eutyroxin-treated rats, rats in the HYD-H and HYD-Y groups presented significantly decreased absolute and relative thyroid weights(*P*<0.01, Figure 4). However, they did not significantly differ between the HYD-H and HYD-Y groups.

### 3.5. Thyroid Function Parameters

**Figure 5:**
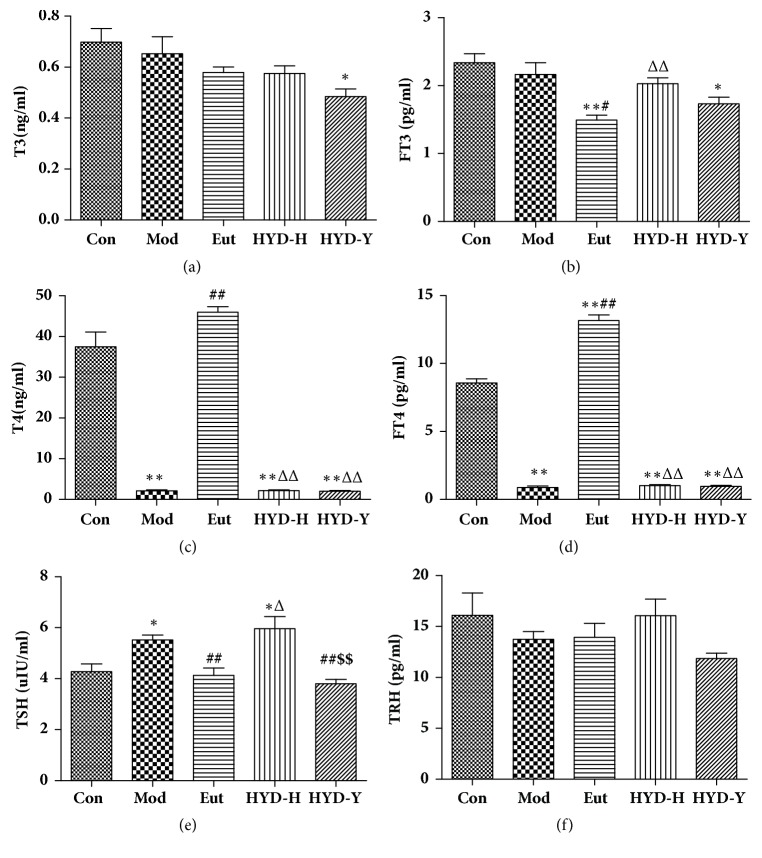
**Serum levels of triiodothyronine (T3), free T3 (FT3), thyroxine (T4), free T4 (FT4), thyroid-stimulating hormone (TSH), and thyrotropin-releasing hormone (THR) in rats in all groups after drug treatment for 28 days.** (a), (b), (c), and (d) Both HYD-H and HYD-Y had no effect on the levels of T3, FT3, T4, and FT4 in sera of goiter rats. (e) Elevated level of TSH in sera of goiter rats could be efficiently decreased by the administration of HYD-Y. (f) There was no difference among all groups in the serum level of TRH. **∗**P<0.05 and **∗∗**P<0.01 versus Con; #P<0.05 and ##P<0.01 versus Mod; ΔP<0.05 and ΔΔPP<0.01 versus Eut; $P<0.05 and $$P<0.01 versus HYD-H, by one-way analysis of variance.

After drug treatment for 28 days, serum levels of T3 and FT3 did not significantly differ among the Con, Mod, and HYD-H groups. HYD-Y treatment markedly decreased the T3 and FT3 levels (*P*<0.05, Figures 5(a) and 5(b)); however, there was no significant difference among the model, HYD-H, and HYD-Y groups. Serum levels of T4 and FT4 in rats in the Mod, HYD-H, and HYD-Y groups were significantly lower than those in the Con group were (*P*<0.01, Figures 5(c) and 5(d)). However, there was no significant difference among these levels in the Mod, HYD-H, and HYD-Y groups. Serum levels of TSH in the Mod and HYD-H groups were significantly higher than those in the Con group were, and there was no difference between serum TSH levels in the Mod and HYD-H groups (*P*<0.01, Figure 5(e)). However, the serum TSH level in the HYD-Y group was significantly lower than that in the Mod group was (*P*<0.01, Figure 5(e)). TRH levels did not differ among the groups (Figure 5(f)).

**Figure 6:**
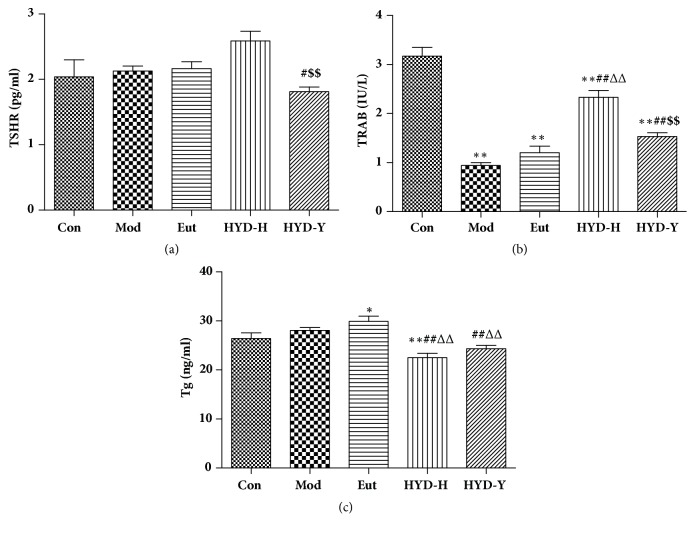
**Serum levels of thyrotropin-releasing hormone receptor (TSHR), TSHR antibody (TRAb), and thyroglobulin (Tg) in rats in all groups after drug treatment for 28 days.** (a) HYD-Y could significantly decrease the level of TSHR in sera to compare with the other treatments. (b) Reduced level of TRAb in sera of goiter rats could be efficiently increased by the administration of HYD-H and HYD-Y. (c) Both HYD-H and HYD-Y could efficiently decrease the level of Tg in sera of goiter rats. **∗**P<0.05 and **∗∗**P<0.01 versus Con; #P<0.05 and ##P<0.01 versus Mod; ΔP<0.05 and ΔΔPP<0.01 versus Eut; $P<0.05 and $$P<0.01 versus HYD-H, by one-way analysis of variance.

The serum level of TSHR in the HYD-Y group was significantly lower than that in the Mod group was (*P*<0.01, Figure 6(a)). Serum levels of the TRAb in the Mod, HYD-H, and HYD-Y groups were significantly lower than those in the Con group were (*P*<0.01, Figures 5(a) and 5(b)). Serum levels of TRAb in the HYD-H and HYD-Y groups were significantly higher than those in the Mod group were (*P*<0.01, Figure 6(b)). The serum level of TRAb in the HYD-H group was significantly higher than that in the HYD-Y group was (P<0.01, Figure 6(b)). There was no difference between serum Tg levels in the Con and Mod groups, and levels in the HYD-H and HYD-Y groups did not differ between each other but levels in both groups were significantly lower than those in the Mod group were (*P*<0.05, Figure 6(c)).

### 3.6. Serum Levels of MDA, SOD, and GSH-PX

**Figure 7:**
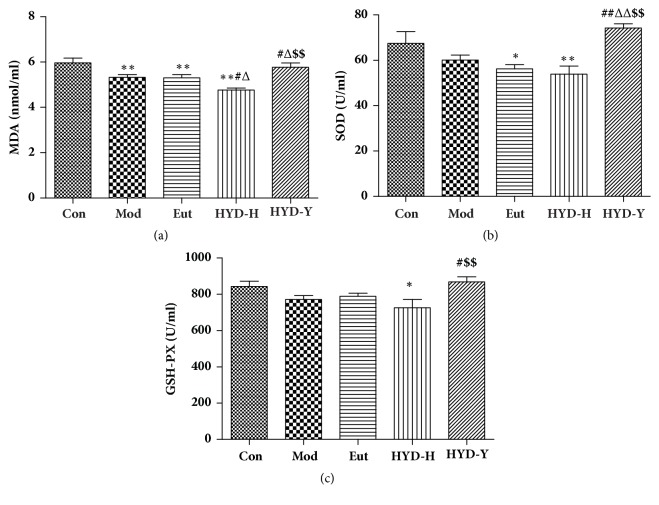
**Serum Levels of malondialdehyde (MDA), superoxide dismutase (SOD), and glutathione peroxidase in rats in all groups after drug treatment for 28 days.** (a) The serum levels of MDA of goiter rats could be efficiently increased by the administration of HYD-H and HYD-Y. (b) and (c) The serum levels of SOD and GSH-PX of goiter rats could be efficiently increased by the administration of HYD-Y. **∗**P<0.05 and **∗∗**P<0.01 versus Con; #P<0.05 and ##P<0.01 versus Mod; ΔP<0.05 and ΔΔPP<0.01 versus Eut; $P<0.05 and $$P<0.01 versus HYD-H, by one-way analysis of variance.

Serum levels of MDA, SOD, and GSH-PX in the HYD-H group were significantly lower than those in the Con group were, whereas they were significantly higher in the HYD-Y group than in the Mod and HYD-H groups (*P*<0.05, Figure 7).

### 3.7. mRNA Levels of Tg, Thyroid Peroxidase (TPO), Sodium Iodide Symporter (NIS), B-Cell Lymphoma-2 (Bcl-2), and Fas

**Figure 8:**
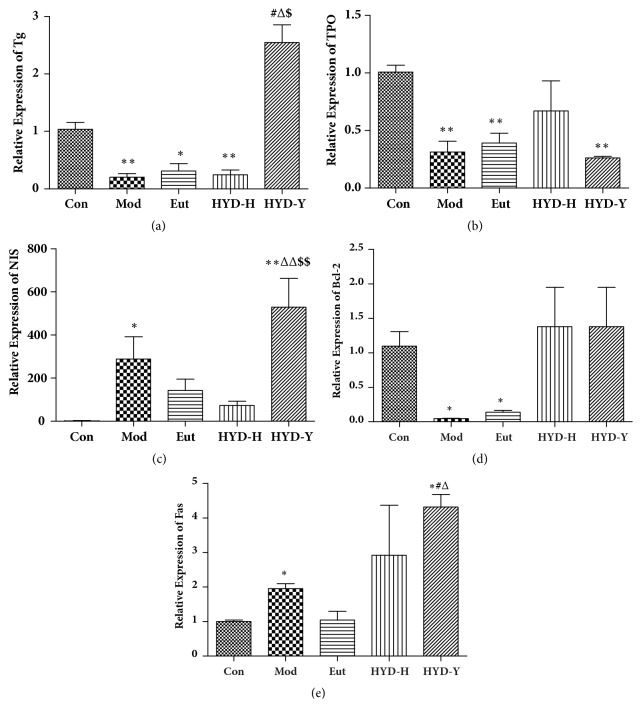
**Real-time polymerase chain reaction (PCR) analysis of thyroglobulin (Tg), thyroid peroxidase (TPO), sodium iodide symporter (NIS), B-cell lymphoma-2 (Bcl-2), and Fas after drug treatment for 28 days.** (a) HYD-Y increased the expression of Tg in goiter rats. (b) Both HYD-H and HYD-Y had no effect on the expression of TPO in goiter rats. (c) HYD-Y increased the expression of NIS. (d) Both HYD-H and HYD-Y had no effect on the expression of TPO in goiter rats. (e) HYD-Y increased the expression of Fas in goiter rats. **∗**P<0.05 and **∗∗**P<0.01 versus Con; #P<0.05 and ##P<0.01 versus Mod; ΔP<0.05 and Δ**Δ**PP<0.01 versus Eut; $P<0.05 and $$P<0.01 versus HYD-H, by one-way analysis of variance.

Real-time PCR analysis showed that mRNA levels of Tg, TPO, and Bcl-2 in thyroid tissues of model rats group were lower than those in control rats were, whereas HYD-Y administration enhanced the expression of Tg and Bcl-2. mRNA levels of NIS and Fas in thyroid tissues of rats in the Mod and HYD-Y groups were significantly higher than those in the Con group were (*P*<0.05, Figure 8).

### 3.8. Histological Observations

**Figure 9:**
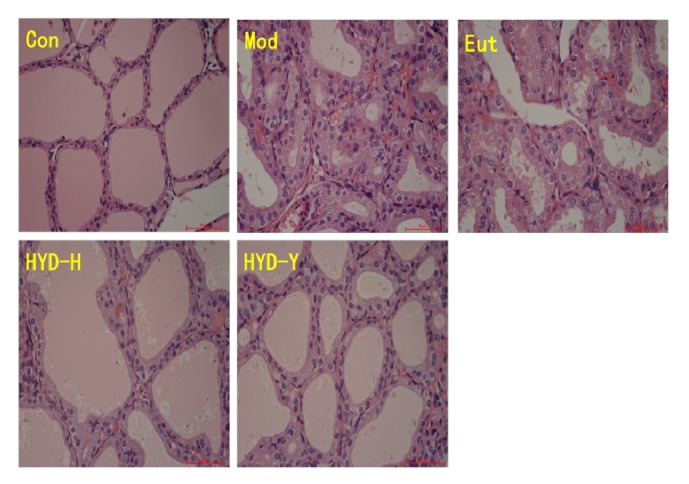
**Histological observations of the thyroid tissues in different groups (H&E staining) after drug treatment for 28 days.** Thyroid tissues in the Mod and Eut groups showed diffuse hyperplasia and hypertrophy of follicular epithelial cells with an irregular arrangement, and follicular secretions were significantly lower in these groups than in the normal Con group. The administration of HYD-H and HYD-Y prevented hyperplasia and hypertrophy of follicular epithelial cells.

Structurally, thyroid tissues in the Mod and Eut groups showed diffuse hyperplasia and hypertrophy of follicular epithelial cells with an irregular arrangement, and follicular secretions were significantly lower in these groups than in the normal Con group. However, the administration of HYD-H and HYD-Y prevented hyperplasia and hypertrophy of follicular epithelial cells, but no significant differences were observed between these two treatment groups (Figure 9).

## 4. Discussion

In this study, we investigated the therapeutic effects of two different species of* Sargassum* coadministered with GC in HYD to rats with PTU-induced goiter and attempted to explore the possible underlying mechanisms.

PTU, as an antithyroid agent, interferes with iodide oxidation and inhibits tyrosyl iodination to prevent thyroid hormone synthesis. In our study, PTU administration for 2 weeks significantly reduced serum levels of T4, rectal temperature, and body weight, and increased serum level of TSH of model rats. In addition, based on our previous report [7, 11], we believe that the goiter model was established. At the end of the study, rats in the Mod group presented with significantly enlarged thyroid glands and hypothyroidism characterized by low metabolism, body weight, and rectal temperature as well as fatigue and unresponsiveness, which further confirmed successful model establishment. Euthyroxin contains levothyroxine sodium that can be directly used by the body. In this study, although Euthyroxin significantly increased serum thyroid level and prevented a decline in metabolism due to hypothyroidism, it did not significantly prevent hyperplasia and hypertrophy of follicular epithelial cells. However, HYD-H and HYD-Y inhibited hyperplasia and hypertrophy of follicular epithelial cells.

T3, T4, FT3, FT4, and TSH are the main indicators of thyroid function. In our study, all rats except for the Con group, were administered PTU every 2 days to maintain the goiter during the treatment. Serum levels of T4 and FT4 in the HYD-H and HYD-Y groups indicated that HYD did not prevent the PTU-induced inhibition of tyrosyl iodination. TSH is required for thyroid hormone synthesis and release. It affects the expression of NIS, TPO, and Tg; alters the priority of tyrosyl iodination and hormone synthesis; and promotes the rapid internalization of Tg by thyrocytes through interconnected pathways [12]. It also stimulates TSHR and TRAb expression. Our study showed that HYD-Y prevent PTU-induced elevation in serum TSH level.

TSHR and TRAb are implicated in the pathogenesis of Grave's disease. TSH binds to and activates TSHR, resulting in hyperthyroidism and goiter. TRAb is divided into activating (TSAbs), blocking (TBAbs), or neutral (N-TRAbs), which affect the onset of diffuse goiter and cell proliferation [13–16]. Our data showed that serum TSHR levels in the HYD-Y group were significantly lower than those in the other groups were. HYD-H and HYD-Y prevented PTU-induced decrease in serum TRAb levels, and HYD-H treatment was more effective than HYD-Y was. The serum Tg level, which usually increases in nontoxic goiter and is related to thyroid size, is also a marker of thyroid cancer [17]. We observed that HYD-H and HYD-Y decreased serum Tg levels, which correlated with the results of the relative thyroid weight and histological observations.

NIS, TPO, and Tg are critical to the synthesis of thyroid hormones. Genetic abnormalities in any of these substances can cause diseases such as goiter, hypothyroidism, and autoimmune manifestations [18–20]. We observed that the mRNA expression of TPO and Tg was low in the Mod group, whereas the mRNA expression of NIS was high. PTU inhibited tyrosyl iodination, thereby leading to TPO and Tg underexpression. The overexpression of NIS might stimulate the concentration of iodine for the synthesis of thyroid hormones. HYD-Y significantly increased the mRNA expression of Tg and NIS. However, TPO was still underexpressed after administration of HYD. Finally, the levels of thyroid hormones in the HYD groups were still lower than those in the Con group were.

The preliminary step in thyroid hormone formation is the attachment of iodine to tyrosyl residues in Tg to produce monoiodotyrosine and diiodotyrosine. This process occurs at the apical plasma membrane-follicle lumen boundary and involves hydrogen peroxide (H_2_O_2_), iodide, TPO, and glycosylated Tg. Oxidative stress is common in the thyroid tissue during the utilization of H_2_O_2_ for thyroxine synthesis. Therefore, thyroid hormone synthesis depends on the oxidative and antioxidative status of the organism. Hypothyroidism and hyperthyroidism could modify the oxidant-antioxidant balance in the serum and tissues [21].There are also numerous reports on oxidative stress and thyroid states, especially thyroid cancer, although the mechanisms are still unknown [22–25]. In this study, HYD-H and HYD-Y affected the oxidative and antioxidative status of the organism by decreasing or increasing the serum levels of MDA in goiter rats. Additionally, HYD-Y also increased the serum levels of SOD and GSH-PX in goiter rats.

Apoptosis is a normal physiological phenomenon, and disturbance of its regulation triggers the pathogenesis of many diseases [13, 26]. We analyzed the mRNA expression of Bcl-2 and Fas and both HYD-H and HYD-Y inhibited PTU-induced increase in the mRNA expression of Bcl-2. Moreover, Fas mRNA expression in the HYD-Y group was high.

## 5. Conclusions

**Figure 10:**
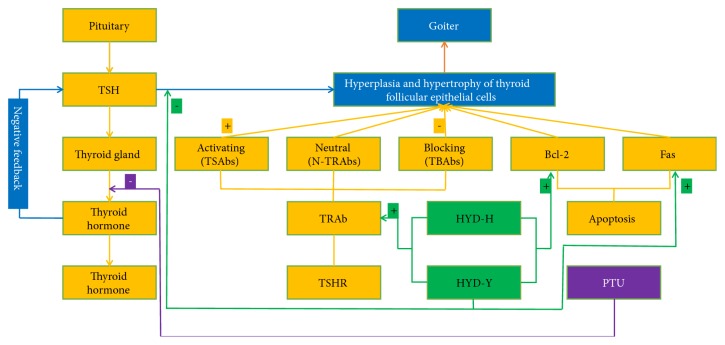
**The regulation of Haizao Yuhu Decoction (HYD) for goiter.** PTU inhibited thyroid hormone synthesis. The decreased serum levels of thyroid hormones negative feedback stimulated the secretion of TSH; then TSH stimulated hyperplasia and hypertrophy of thyroid follicular epithelial cells. Both HYD-H and HYD-Y could stimulate the expression of Bcl-2 and elevate the serum level of TRAb to inhibit the hyperplasia of follicular cells. HYD-Y also presented the function of altering the level of TSH in serum and the expression of Fas mRNA.

**Figure 11:**
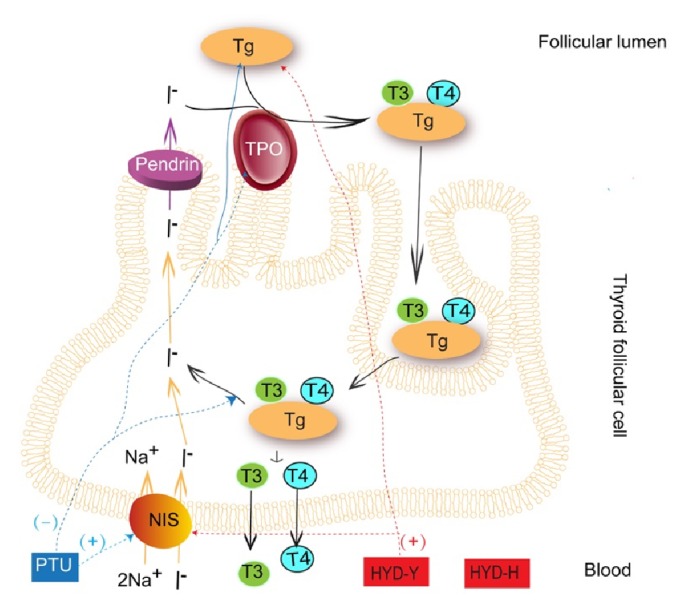
**The regulation of Haizao Yuhu Decoction (HYD) and propylthiouracil (PTU) in thyroid hormone synthesis.** PTU inhibited thyroid hormone synthesis by interfering with iodide oxidation. PTU inhibited the expression of TPO and Tg, stimulated the expression of NIS. HYD-Y stimulated the expression of Tg and NIS. It seemed that HYD-H had no effects on thyroid hormone synthesis.

In conclusion, results of this study showed that HHZ or YQC coadministered with GC at four times the high-dose limit specified by the Chinese Pharmacopoeia in HYD had antigoiter effects. These effects were mediated by its prevention of PTU-induced changes in tissue pathology and relative thyroid weight ratios by stimulating the expression of Bcl-2 and elevating the serum level of TRAb to inhibit the hyperplasia of follicular cells (Figure 10). However, neither HYD-H nor HYD-Y increased the PTU-induced decrease in serum levels of thyroid hormones. Additionally, there were some differences between HYD-H and HYD-Y. HYD-Y decreased serum TSH and TSHR levels and increased Tg and NIS mRNA expressions (Figures 10 and 11). Only HYD-Y increased the serum levels of SOD and GSH-PX in goiter rats.

In the study, we determined the contents of several main active components in the two kinds of HYD: liquiritin, glycyrrhizic acid, hesperidin, peimine, peiminine, osthole, forsythin, and ferulic acid. They all have a variety of pharmacological activities. Liquiritin, glycyrrhizic acid, hesperidin, osthole, and ferulic acid were reported to possess anticancer [27–35], antioxidative [36–44], and anti-inflammatory [30, 37, 44–47] properties. Forsythin was found to possess antioxidant and antibacterial activity [48, 49]. Peimine and peiminine have been reported to have anti-inflammatory properties [50–53], and peiminine suppressed the proliferation and growth of cancer cell by inducing autophagic cell death [54]. Liquiritin exerted anticancer effects by inducing the apoptosis of cancer cells [27, 28]. Glycyrrhizic acid was reported to possess anticancer effects through targeted apoptotic pathways and to control angiogenesis in cancer tissues [29, 30]. Hesperidin exhibited anticancer effects by alleviating oxidative stress [40], inducing apoptosis [31, 32], and inhibiting cell migration and invasion [32]. Osthole inhibited proliferation of cancer cells through the induction of apoptosis [55]. Ferulic acid might act as an anticancer drug by inhibiting autophagy, inducing cell cycle arrest [34], and inducing apoptosis [35]. Phytochemicals had been shown to have either beneficial or detrimental effects on thyroid function [56]. However, the role of the above substances except for hesperidin in thyroid diseases has rarely been reported. Hesperidin, a natural bioflavonoid, was reported to inhibit thyroid functions [57] by inhibiting thyroperoxidase and deiodinase activity [58]. In our study, HYD inhibited the hyperplasia of follicular cells, which might be related to the apoptosis induction and inhibition of proliferation induced by the above substances. However, the dose-effect relationship and the specific mechanism remain unclear. In our study, the levels of hesperidin, peimine, peiminine, osthole, and forsythin were different in HYD-H and HYD-Y. These differences might be the reasons for their different effects on the levels of TSH, TSHR, SOD, and GSH-PX, but the detailed mechanism still needed further studies.

We demonstrated that the two species of* Sargassum* coadministered with GC in HYD exerted antigoiter effects on PTU-induced goiter in rats. Apoptosis might the most important mechanisms. However, the detailed underlying mechanisms remain unclear, and further studies are required to elucidate the material basis and potential side effects of HYD-H and HYD-Y.

## Figures and Tables

**Table 1:** Compositions of HYD.

**Latin name**	**Chinese name**	**Daily adult dose (g)**	**Production and lot number**
***Sargassum pallidum *(Turn.) C.Ag./**	Hai Hao Zi (HHZ)	48	Hainan (China), 20160101
***Sargassum fusiforme *(Harv.) Setch.**	Yang Qi Cai (YQC)		Shandong (China), 20160101
***Glycyrrhiza uralensis* Fisch.**	Sheng Gan Cao (SGC)	40	Ningxia (China), 20160101
***Pinellia ternata *(Thunb)Breit**	Fa Ban Xia (FBX)	9	Guizhou (China), 20160101
***Fritillaria thunbergii* Miq.**	Zhe Bei Mu (ZBM)	9	Zhejiang (China), 20160101
***Laminaria japonica *Aresch.**	Hai Dai (HD)	9	Shandong (China), 20160101
***Forsythia suspensa* (Thunb.) Vahl**	Lian Qiao (LQ)	9	Shanxi (China), 20160101
***Ligusticum chuanxiong* Hort.**	Chuan Xiong (CX)	9	Sichuan (China), 20160101
***Angelica pubescens* Maxim. *f. biserrata Shanet Yuan***	Du Huo (DH)	9	Sichuan (China), 20160101
***Laminaria japonica* Aresch.**	Kun Bu (KB)	9	Fujian (China),1311421
***Citrus reticulata* Blanco**	Qing Pi (QP)	9	Sichuan (China),1509222
***Citrus reticulata *Blanco**	Chen Pi (CP)	9	Sichuan (China),1608005
***Angelica sinensis* (Oliv) Diels.**	Dang Gui (DG)	9	Gansu (China),1604043

**Table 2:** Quantitative determination of several main active components in HYD (mg/ml).

	HYD-H	HYD-Y
**Liquiritin**	0.0503±0.0032	0.0535±0.0035
**Glycyrrhizic Acid**	0.351±0.0244	0.3749±0.0302
**Hesperidin**	1.9602±0.0564	2.4639±0.021*∗∗*
**Peimine**	0.0062±0.0003	0.0052±0.0001*∗∗*
**Peiminine**	0.0058±0.0002	0.0049±0.0001*∗∗*
**Osthole**	0.0521±0.0005	0.0546±0.0013*∗*
**Forsythin**	0.2564±0.0088	0.307±0.0036*∗*
**Ferulic Acid**	1.7312±0.0414	1.6106±0.0329

Quantitative data are expressed as mean ± standard deviation. **∗***P*<0.05 and *∗∗P*<0.01, by one-way analysis of variance.

**Table 3:** The primer sequences of relative genes.

Gene	Forward	Reverse
*β*-actin	5′-GCAGTTGGTTGGAGCAA-3′	5′-ATGCCGTGGATACTTGGA-3′
TPO	5′-GGAAGCAGATGAAGGCTCTG-3′	5′-CGGTGTTGTCACAGATGACC-3′
Tg	5′-TTGATGCCAGTTCTCCTGTG-3′	5′-TGAGGACACTGTGACGAAGC-3′
NIS	5′-CCGGATCAACCTGATGGACT-3′	5′-GCCACATAGCGCTGTACCTG-3′
Fas	5′-ATCATGGCTGTCCTGCCTCT-3′	5′-TCACGAACGCTCCTCTTCAA-3′
Bcl-2	5′-CACGGTGGTGGAGGAACTCT-3′	5′-TCCACAGAGCGATGTTGTCC-3′

## Data Availability

The data used to support the findings of this study are available from the corresponding author upon request.
